# Early Response of Protein Quality Control in Gills Is Associated with Survival of Hypertonic Shock in Mozambique tilapia

**DOI:** 10.1371/journal.pone.0063112

**Published:** 2013-05-14

**Authors:** Cheng-Hao Tang, Tsung-Han Lee

**Affiliations:** 1 Institute of Marine Biotechnology, National Dong Hwa University, Pingtung, Taiwan; 2 National Museum of Marine Biology and Aquarium, Pingtung, Taiwan; 3 Department of Life Sciences, National Chung Hsing University, Taichung, Taiwan; 4 Department of Biological Science and Technology, China Medical University, Taichung, Taiwan; University of Iowa, United States of America

## Abstract

The protein quality control (PQC) mechanism is essential for cell function and viability. PQC with proper biological function depends on molecular chaperones and proteases. The hypertonicity-induced protein damage and responses of PQC mechanism in aquatic organisms, however, are poorly understood. In this study, we examine the short-term effects of different hypertonic shocks on the levels of heat shock proteins (HSPs, e.g., HSP70 and HSP90), ubiquitin-conjugated proteins and protein aggregation in gills of the Mozambique tilapia (*Oreochromis mossambicus*). Following transfer from fresh water (FW) to 20‰ hypertonicity, all examined individuals survived to the end of experiment. Moreover, the levels of branchial HSPs and ubiquitin-conjugated proteins significantly increased at 3 and 24 h post-transfer, respectively. Up-regulation of HSPs and ubiquitin-conjugated proteins was sufficient to prevent the accumulation of aggregated proteins. However, the survival rate of tilapia dramatically declined at 5 h and all fish died within 7 h after direct transfer to 30‰ hypertonicity. We presumed that this result was due to the failed activation of gill PQC system, which resulted in elevating the levels of aggregated proteins at 3 and 4 h. Furthermore, in aggregated protein fractions, the amounts of gill Na^+^/K^+^-ATPase (NKA) remained relatively low when fish were transferred to 20‰ hypertonicity, whereas abundant NKA was found at 4 h post-transfer to 30‰ hypertonicity. This study demonstrated that the response of PQC in gills is earlier than observable changes in localization of ion-secreting transport proteins upon hypertonic challenge. To our knowledge, this is the first study to investigate the regulation of PQC mechanism in fish and characterize its important role in euryhaline teleost survival in response to hypertonic stress.

## Introduction

Environmental stresses cause numerous perturbations that are detrimental to cellular homeostasis and physiological function. Compensatory responses induced by diverse stresses are essential for cell survival under adverse conditions. The mechanisms of induced cellular responses are regulated by many molecular processes that aid cell adaptation to various stresses [1]. Furthermore, proteins are the molecules indeed possess the biological function to carry out diverse mechanisms responsible for cellular function. The expression and maintenance of functional proteins depends on more than transcription and translation [2]. Chaperones and proteases mediate protein quality control (PQC), prevent protein aggregation, and maintain cell viability in many species under stressful conditions, from unicellular organisms to plants and animals [2]–[5].

Aquatic organisms are naturally confronted with environmental changes, including salinity. Changes in environmental salinity can lead to osmotic stress, the ability of an organism to successfully adapt to the osmotic challenge is complex and involves many regulatory aspects [6]–[7]. The fish gill is a multifunctional organ in direct contact with the external environment [8], [9]. Therefore, alteration of ambient salinity would seriously affect the homeostasis of branchial cells. Euryhaline teleosts are an excellent model to study osmoregulatory mechanisms *in vivo*. The systemic osmoregulatory mechanisms have been investigated in fish for many years. These investigations, however, primarily focused on ion transport in major osmoregulatory tissues [8], [10]–[13]. A considerable up-regulation of branchial Na^+^/K^+^-ATPase (NKA), Na^+^K^+^/2Cl^−^ cotransporter (NKCC) and cystic fibrosis transmembrane conductance regulator (CFTR) is required for euryhaline fish to adapt to hypertonic environments [10], [12], [14].

**Figure 1:**
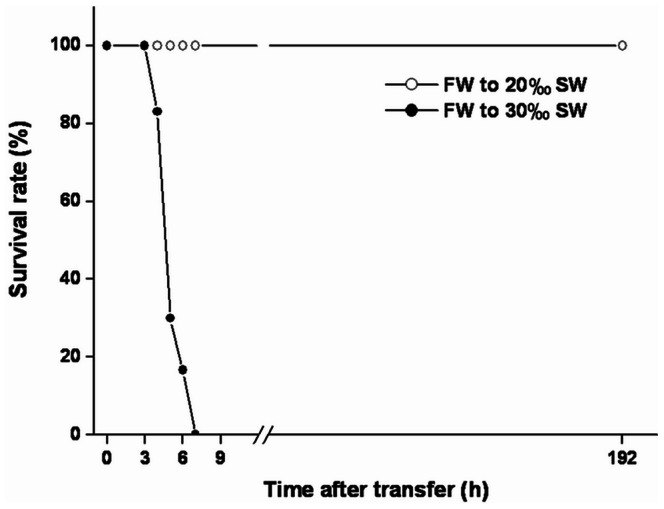
Survival rates (%) of fresh water (FW)-acclimated tilapia transferred to different hypertonic conditions. Open and closed circles indicated that FW-acclimated tilapia were directly transferred to hypertonic conditions of 20‰ and 30‰ seawater (SW), respectively. n = 30 for each experiment.

Mozambique tilapia (*Oreochromis mossambicus*) have been widely used to study osmoregulatory mechanisms because they have great euryhalinity [9], [15]–[16]. Upon hypertonic challenge, tilapia can survive direct transfer from fresh water (FW) to 20‰ seawater (SW) (Fig. 1). However, unlike killifish, *Fundulus heteroclitus*; pufferfish, *Tetraodon nigrovirids*; eel, *Anguilla anguilla* or *A. japonica*) [7], [9], [17], tilapia can not survive direct transfer from FW to near full-strength SW (≥30‰) [7], [9], [16], [18]; all tilapia died shortly after direct transfer from FW to 30‰ SW (Fig. 1).

Although there were some reports of heat shock protein (HSP) responses and ubiquitinated proteins in aquatic organisms [6], [19]–[21], the mechanism of PQC in aquatic organisms upon osmotic stress has not been studied in depth. The present study explored changes in the PQC in fish gills following salinity stress by comparing the responses in FW tilapia transferred to either 20‰ or 30‰ hypertonic environments. Our study suggests that activation of the PQC mechanism limits protein aggregation and that protein aggregation may contribute to mortality if not properly mitigated. To our knowledge, this is the first study to provide *in vivo* evidence for the PQC playing an important role in osmoprotection in a euryhaline organism.

## Materials and Methods

### Experimental Animals and Environments

Tilapia 6–10 g in weight and 5–8 cm in length were obtained from laboratory stocks. Fish were reared in a tank containing 300 L of aerated local tap water (fresh water (FW)) ([Na^+^] 2.6 mM; [Cl^−^] 0.2 mM; [Ca^2+^] 0.58 mM) at 26±1°C with a daily 12-h photoperiod. Different hypertonic environments (20‰ or 30‰ seawater; SW) were prepared from local tap water with proper amounts of “Instant Ocean” synthetic sea salt (Aquarium Systems, Mentor, OH, USA). The waters were continuously circulated through fabric-floss filters, and the environmental osmolalities were measured by the Wescor 5520 vapro osmometer (Logan, Utah, USA). Fish were fed daily with commercial pellets except 48 h prior to the experiments. The facilities and protocols for the experimental fish were approved by the Animal Care and Utility Committee of National Chung Hsing University (approval no. 96-48).

### Acclimation Procedures and Survival Rate

Five FW tilapia were transferred to a tank with 50 liters of aerated FW for one week before acclimation experiments to avoid crowded stress. Experiments began when tilapia were directly transferred to 50 liters recirculation aquariums containing either 20‰ or 30‰ hypertonic medium. For the experiments of survival rate, 30 fish were transferred from FW to different hypertonic conditions, and the number of dead fish was recorded every hour. For other experiments, fish were sampled at the indicated times, and the studied tissues were removed. For Western blot analysis and protein aggregation experiments, tissues were immediately frozen in liquid nitrogen and subsequently stored at −80°C.

### Antibodies

The primary antibodies used in the present study included (1) anti-Na^+^/K^+^-ATPase (NKA), a rabbit monoclonal antibody (ab76020; Abcam, Cambridge, MA, UK) raised against the N-terminus of human NKA α-subunit; (2) anti-cystic fibrosis transmembrane conductance regulator (CFTR), a mouse monoclonal antibody to human CFTR (R&D Systems, MN, USA) that was raised against a carboxy-terminal sequence of human CFTR and has been successfully used in several fish species [22]–[24]; (3) anti-Na^+^/Cl^−^ cotransporter (NCC)/Na^+^/K^+^/2Cl^−^ cotransporter (NKCC), a mouse monoclonal antibody (T4; Developmental Studies Hybridoma Bank, Iowa City, IA, USA) raised against the C-terminus of human NKCC, which has been demonstrated to recognize apical NCC and basolateral NKCC in mitochondrion-rich cells of tilapia, respectively [14], [24]; (4) anti-HSP70, a mouse monoclonal antibody (H 5147; Sigma, St. Louis, MO, USA) generated by immunization with purified bovine brain HSP70; (5) anti-HSP90, a rabbit polyclonal antibody (#4874; Cell Signaling Technology, Beverly, MA, USA) corresponding to human HSP90; (6) anti-ubiquitin, a rabbit polyclonal antibody (#3933; Cell Signaling Technology) corresponding to the N-terminus of the human ubiquitin protein that detects ubiquitin, polyubiquitin and ubiquitinated proteins and (7) anti-β-actin, a monoclonal antibody (ab8226, Abcam) against residues 1–100 of human β-actin. The secondary antibodies for Western blot analyses were alkaline phosphatase-conjugated goat anti-rabbit or anti-mouse IgG (Chemicon, Temecula, CA, USA), and for immunofluorescent staining were DyLight 488 or 549 conjugated goat anti-rabbit or anti-mouse IgG (Jackson Immunoresearch, West Baltimore Pike, PA, USA).

### Whole-mount Double Immunofluorescent Staining

Whole-mount immunofluorescent staining was carried out as described by Tang and Lee [25]. The first pair of gills was excised, and the gill filaments were removed from the gill arch and immediately fixed in 0.5% glutaraldehyde and 4% paraformaldehyde in phosphate-buffered saline (PBS) for 1 h at 4°C. After washing with 0.2% Triton X-100 in PBS (PBST), the gill filaments were post-fixed and permeated with 70% ethanol for 10 min at −20°C. The gill filaments were rinsed with PBST and then incubated in PBST containing 5% bovine serum albumin (Sigma, St. Louis, MO, USA) for 1 h at room temperature (25–28°C) to reduce non-specific binding. For double immunofluorescent staining, the gill filaments were first incubated at room temperature for 2 h with the primary rabbit monoclonal antibody to NKA (ab76020). The gill filaments were washed several times with PBST and subsequently labeled with DyLight 549-conjugated goat anti-rabbit secondary antibody (Jackson Immunoresearch) at room temperature for 2 h. After the first staining, the gill filaments were washed several times with PBST to proceed to the second staining. The gill filaments were subsequently incubated with primary mouse monoclonal anti-NCC/NKCC or anti-CFTR antibodies at 4°C for 12 h followed by labeling with Dylight 488-conjugated goat anti-mouse secondary antibody (Jackson Immunoresearch) at room temperature for 2 h. The samples were then washed with PBST, mounted with a coverslip, and observed with a confocal laser microscope (FV 1000, Olympus, Tokyo, Japan). The 488-nm argon-ion laser and the 546-nm helium-neon laser were used for DyLight 488 and 549, respectively.

### Cell Protein Fractionation and Isolation of Aggregated Proteins

The procedures of cell protein fractionation and isolation of aggregated proteins were performed according to published studies [26]–[28]. The studied tissues were homogenized in chilled extraction buffer containing 0.1% Triton X-100, 60 mM PIPES, 1 mM EDTA, 1 mM ethylene glyco-bis (-aminoethyl ether)-N,N,N,N-tetraacetic acid and 100 mM NaCl. In addition, 40 µl of a proteinase inhibitor cocktail (Roche, Mannheim, Germany) was added for each mL of chilled extraction buffer. Homogenization was performed in 2 ml tubes with a Polytron PT1200E (Lucerne, Switzerland) at maximal speed for 20 strokes. The homogenate was centrifuged at 680 *g* for 10 min at 4°C to pellet nuclei and large cellular fragments. The supernatant was assigned to the total cell lysates for the following Western blot analyses. The resulting supernatant (total cell lysate) was centrifuged at 35,000 *g* for 14 min at 4°C to separate the Triton-soluble and insoluble protein fractions. Aggregated proteins were isolated by differential centrifugation. The Triton-insoluble fraction was twice resuspended in extraction buffer, sonicated, and pelleted at 17,000 *g* for 30 min at 4°C. The resultant pellet was again resuspended in extraction buffer, sonicated, and pelleted at 5,000 *g* for 30 min at 4°C. The pellet consisting of aggregated proteins was resuspended in extraction buffer (aggregated protein fraction) and stored at −80°C. Protein concentrations of total cell lysates and aggregated protein fractions were determined with a BCA Protein Assay Kit (Pierce, Hercules, CA, USA) using bovine serum albumin (BSA, Pierce) as a standard.

### Western Blot Analysis

Gill proteins were heated in sample buffer at 90°C for 10 min for detection of HSPs in total cell lysates or at 37°C for 30 min for detection of NKA in aggregated protein fractions. The samples were separated by electrophoresis on sodium dodecyl sulfate (SDS) containing 8% polyacrylamide gels for detection of HSPs and NKA. For analysis of ubiquitinated proteins, the gill samples were mixed with appropriate amounts of sample buffer and separated by electrophoresis on SDS containing 12% polyacrylamide gels (10 µg of protein/lane). The prestained protein molecular weight marker was purchased from Fermentas (SM0671; Hanover, MD, USA). The separated proteins were then transferred to PVDF membranes (0.45 µm pore size) (Millipore, Bedford, MA, USA) by electroblotting. After preincubation for 3 h in PBST buffer containing 5% (wt/vol) nonfat dried milk to minimize nonspecific binding, the blots were incubated at room temperature for 3 h with primary antibody diluted in 1% BSA and 0.05% sodium azide in PBST, washed in PBST, and incubated at room temperature for 2 h with secondary antibody. Blots were developed after incubation with the BCIP/NBT kit (Zymed, South San Francisco, CA, USA). β-actin or Ponceau S total protein stain of blots were used as the loading control for HSPs, or NKA in aggregated fractions and ubiquitinated proteins, respectively. The developed blots were photographed and imported as TIFF files. Immunoreactions were analyzed using a software package (MCID software, Imaging Research, Ontario, Canada). The results were converted to numerical values to compare the relative protein abundance of the immunoreactions.

### Statistical Analyses

The effect of hypertonic stress at various time points was assessed by comparison with the 0 h values, using Dunnett’s test in which salinity effects were detected by one-way ANOVA (*P*<0.05). Statistical significance within the levels of aggregated proteins in studied tissues except gills was determined using Student’s *t*-test (*P*<0.05) for a group data analysis. Values were expressed as means ± standard error of mean (S.E.M).

## Results

### Effects of Acute Hypertonic Challenges (20‰ or 30‰ Seawater, SW) on Survival Rate of Euryhaline Tilapia

During acute transfer to 20‰ SW, all of the experimental tilapia survived until the end of the experiment (Fig. 1; open circle). However, tilapia did not survive more than 7 h after direct transfer from fresh water (FW) to 30‰ hypertonicity (30‰ SW) (Fig. 1; close circle). This result revealed that the critical and acute mechanisms for tilapia adaptation to hypertonic stress were induced in transfer to 20‰ SW but not to 30‰ SW.

### Whole-mount Immunofluorescent Staining of Ion-secreting Related Transporter Proteins in Gills of Tilapia Transferred from FW to 20‰ SW

**Figure 2:**
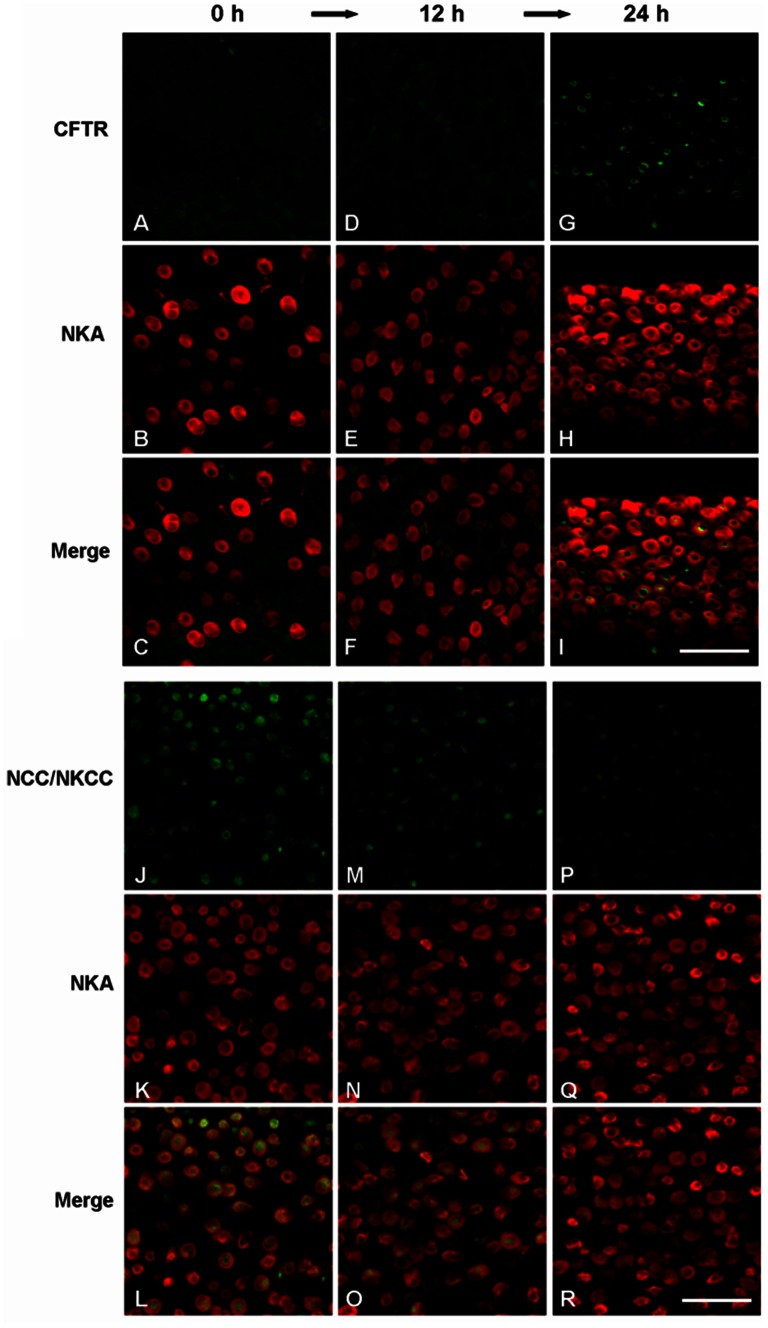
Confocal laser scanning micrographs of whole-mount preparations of gill filaments in tilapia at 0 h (A–C, J–L), 12 h (D–F, M–O) and 24 h (G–I, P–R) after transfer from fresh water (FW) to 20‰ seawater. Gill filaments were double-stained with anti-Na^+^/K^+^-ATPase (NKA) (red) and anti-cystic fibrosis transmembrane conductance regulator (green; A–I) or anti-NCC/NKCC (green; J–R). Scale bars = 40 µm.

It is generally accepted that ion secretion is mediated by apical cystic fibrosis transmembrane conductance regulator (CFTR) Cl^−^ channel and basolateral Na^+^/K^+^/2Cl^−^ cotransporter (NKCC) in gill mitochondrion-rich (MR) cells of fish acclimated to a hypertonic environment [8], [9], [22]–[25]. A time-course transfer experiment was performed to further investigate the time point of induction of branchial CFTR and NKCC in response to hypertonic challenge. Moreover, it has been demonstrated that both apical NCC and basolateral NKCC in MR cells of tilapia can be recognized by the antibody T4 [14], [24]. In fish acclimated to freshwater (0 h), therefore, CFTR and NKCC immunoreactivities were absent (Fig. 2C, 2L), and NCC was present in the apical membrane of MR cells (Fig. 2L). The immunoreactivity of apical CFTR and NCC increased and decreased, respectively, at 24 h post-transfer from FW to 20‰ SW (Fig. 2I, 2R). Moreover, the immunoreactivity of basolateral NKCC was still undetectable at 24 h (Fig. 2R). These revealed that observable changes in ion-secreting mechanisms did not occur within 7 h to cope with hypertonicity-induced damage.

### Evaluation of the Abundance of Gill Heat Shock Proteins (HSPs) and Ubiquitin-conjugated Proteins in Response to Acute Transfer from FW to 20‰ or 30‰ SW

**Figure 3:**
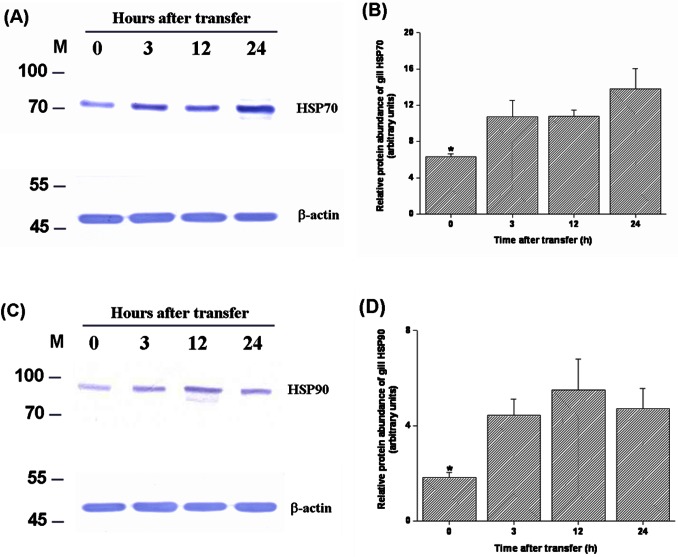
Dynamic changes of protein expression of heat shock protein 70 (HSP70) (A and B) and HSP90 (C and D) in gills of tilapia directly transferred from fresh water to 20‰ seawater. (A and C) Representative Western blots of HSP70 and HSP90 showed a single immunoreactive band at approximately 70 and 90 kDa, respectively. The levels of HSP70 and HSP90 protein significantly elevated during the first 3 h post-transfer and sustained at 24 h after transfer (B and D). β-actin was used as the loading control. The asterisks indicated significant differences (*P*<0.05) compared with the 0 h time-point using Dunnett’s test following a one-way ANOVA. Values are mean ± S.E.M (n = 5). M, marker.

**Figure 4:**
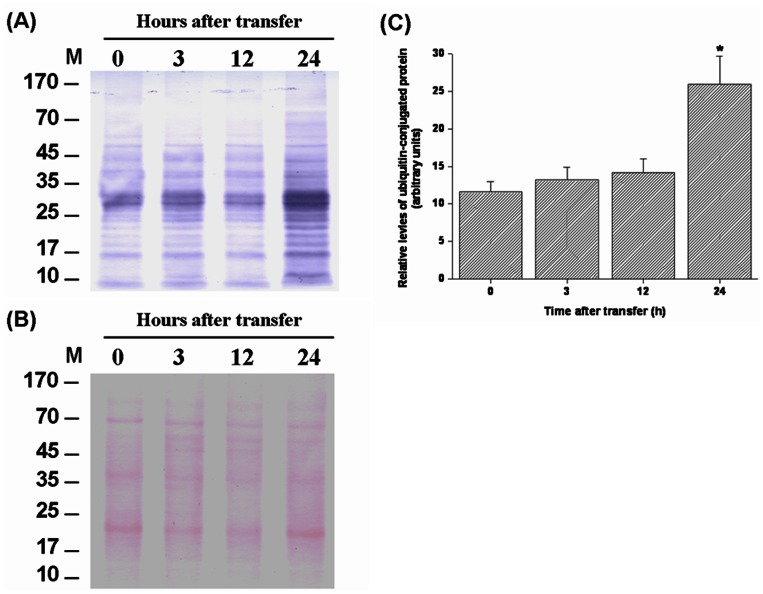
Western blot analysis of the levels of ubiquitin-conjugated proteins in gills of tilapia directly transferred from fresh water (FW) to 20‰ seawater (SW). (A) Ubiquitin-conjugated protein levels were shown as relative values based on lane intensities. (B) Ponceau S total protein stain of membranes was used as the loading control. (C) The levels of ubiquitin-conjugated proteins increased significantly at 24 h post-transfer from FW to 20‰ SW. The asterisk indicated a significant difference (*P*<0.05) compared with the 0 h time-point using Dunnett’s test following a one-way ANOVA. Values are mean ± S.E.M (n = 5). M, marker.

**Figure 5:**
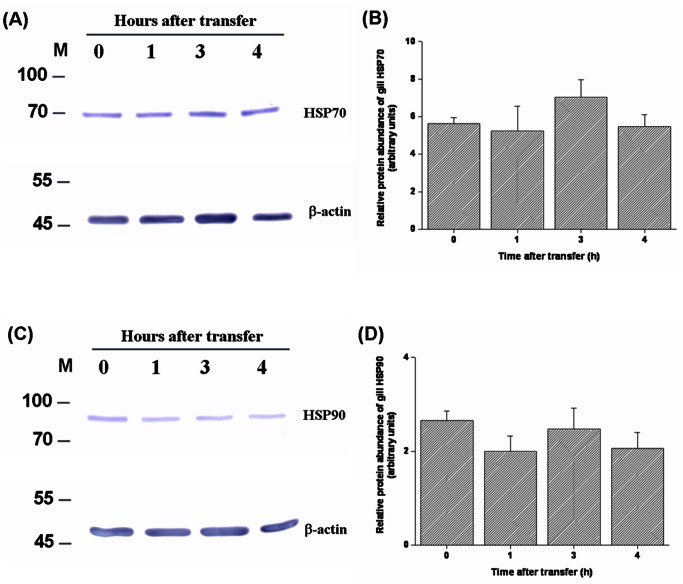
Dynamic changes of protein expression of heat shock protein 70 (HSP70) (A and B) and HSP90 (C and D) in gills of tilapia directly transferred from fresh water to 30‰ seawater (SW). (A and C) Representative Western blots of HSP70 and HSP90 showed a single immunoreactive band at approximately 70 and 90 kDa, respectively. No significant elevation of branchial HSP70 or HSP90 was found in tilapia within 4 h post-transfer from FW to 30‰ (SW) (B and D). β-actin was used as the loading control. The asterisks indicated significant differences (*P*<0.05) compared with the 0 h time-point using Dunnett’s test following a one-way ANOVA. Values are mean ± S.E.M (n = 5). M, marker.

**Figure 6:**
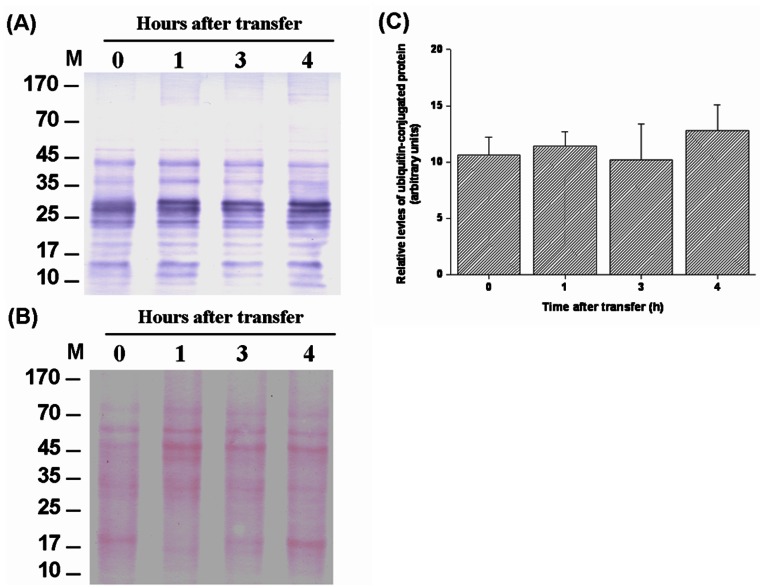
Western blot analysis of the levels of ubiquitin-conjugated protein in gills of tilapia directly transferred from fresh water (FW) to 30‰ seawater (SW). (A) Ubiquitin-conjugated protein levels were shown as relative values based on lane intensities. (B) Ponceau S total protein stain of membranes was used as the loading control. (C) No significant difference was found in the levels of ubiquitin-conjugated proteins when tilapia were transferred from FW to 30‰ SW. Values are mean ± S.E.M (n = 5). M, marker.

**Figure 7:**
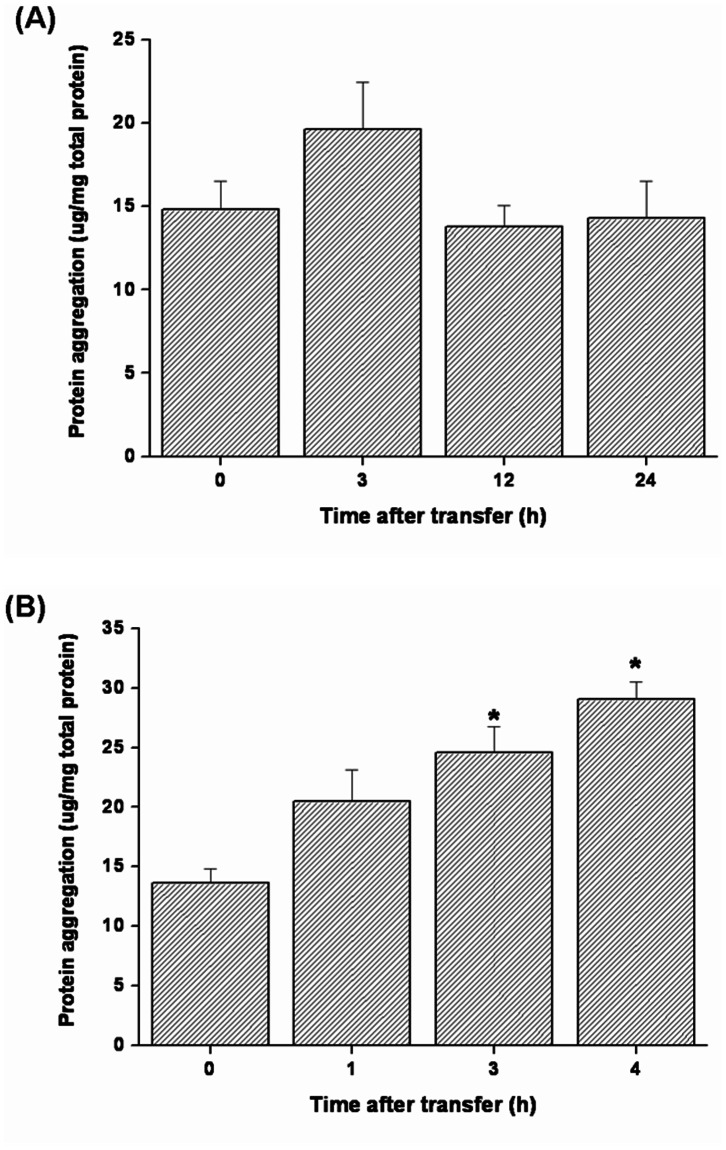
The levels of aggregated proteins in gills of fresh water (FW)-acclimated tilapia acutely exposed to 20‰ (A) or 30‰ seawater (SW) (B). There was no significant difference in the levels of aggregated proteins when tilapia were transferred from FW to 20‰ SW (A). The aggregated protein levels were evidently elevated at 3 and 4 h post-transfer from FW to 30‰ SW (B). The asterisks indicated significant differences (*P*<0.05) compared with the 0 h time-point using Dunnett’s test following a one-way ANOVA. Values are mean ± S.E.M (n = 5).

The levels of HSPs and ubiquitin-conjugated proteins in gills of tilapia transferred to a hypertonic environment (20‰ SW) were determined by Western blot analyses. The data showed that the expression levels of branchial HSP70 and HSP90 increased from 3 h to 24 h after direct transfer of FW tilapia to 20‰ SW (Fig. 3). The hypertonicity-induced up-regulation was also found in ubiquitin-conjugated proteins in tilapia gills at 24 h post-transfer (Fig. 4). In contrast, during transfer to 30‰ SW, a failure in activation of the responses of HSPs and ubiquitin-conjugated proteins was observed (Fig. 5, 6). Importantly, the aggregated proteins were maintained at a low levels (15–18 µg/mg total protein) underlying the elevated responses of HSPs and ubiquitin-conjugated proteins when tilapia were transferred to 20‰ hypertonicity (Fig. 7A). Conversely, the levels of aggregated proteins were significantly elevated at 3 (24.6 µg/mg total protein) and 4 (30 µg/mg total protein) h after tilapia were exposed to a severe hypertonic shock (30‰ hypertonicity) (Fig. 7B).

### Protein Amounts of Na^+^/K^+^-ATPase (NKA) in Aggregated Protein Fractions

**Figure 8:**
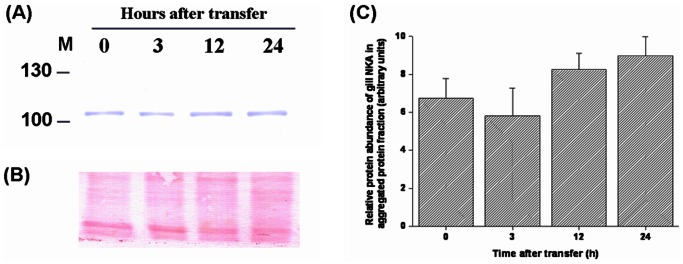
The protein abundance of branchial Na^+^/K^+^-ATPase (NKA) in aggregated protein fractions of fresh water (FW)-acclimated tilapia acutely exposed to 20‰ seawater (SW). (A) Representative Western blots of NKA revealed a single immunoreactive band at approximately 105 kDa. (B) Ponceau S total protein stain of blot. (C) There was no significant difference in the abundance of NKA in aggregated protein fractions of tilapia transferred from FW to 20‰ (SW). Values are mean ± S.E.M (n = 5).

**Figure 9:**
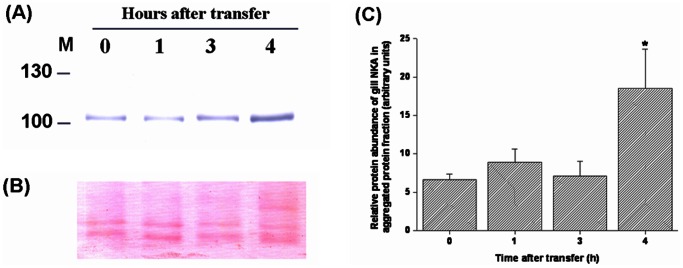
The protein abundance of branchial Na^+^/K^+^-ATPase (NKA) in aggregated protein fractions of fresh water (FW)-acclimated tilapia acutely exposed to 30‰ seawater (SW). (A) Representative Western blots of NKA revealed a single immunoreactive band at approximately 105 kDa. (B) Ponceau S total protein stain of blot. (C) The levels of NKA in aggregated fractions were significantly higher at 4 h post-transfer. The asterisks indicated significant differences (*P*<0.05) compared with the 0 h time-point using Dunnett’s test following a one-way ANOVA. Values are mean ± S.E.M (n = 5).

NKA is important for sustaining intracellular homeostasis and providing the driving force for osmoregulatory systems in fish gills. To study the effect of hypertonic stress on osmoregulatory ability, the abundance of NKA levels in aggregated protein fractions of tilapia gills acutely exposed to 20‰ or 30‰ SW was determined. No significant difference was found when exposed to 20‰ SW for 24 h compared with 0 h (Fig. 8). However, there was a significant 2.8-fold increase of NKA protein levels in aggregated protein fractions at 4 h post-transfer to 30‰ SW (Fig. 9).

## Discussion

Cellular responses induced by environmental stresses are crucial for cell viability under adverse conditions [1]. In the natural environment, salinity is one of the major abiotic factors that strongly influence cellular function and homeostasis in organisms [12], [29]–[33]. Cells must possess diverse regulatory mechanisms to cope with osmotic perturbation and maintain intracellular homeostasis, cellular structures and biological functions [6]–[7], [30], [34]. Euryhaline fish have a series of key mechanisms to maintain cellular and organismal homeostasis during salinity adaptation, therefore, this species is an excellent model to investigate molecular mechanisms that counteract the effects of osmotic challenge [6]–[7], [9], [15]. The fish gills are in direct contact with the environment [8]–[9]. Furthermore, Mozambique tilapia (*Oreochromis mossambicus*) experiences abrupt changes in ambient salinity in its native habitat along the southeast coast of Africa [35]–[36]. Although tilapia possesses the great euryhalinity and successfully survived transfer from fresh water (FW) to 20‰ seawater (SW), all examined tilapia died shortly after direct transfer from FW to 30‰ SW (Fig. 1) [37]. With this regard, the objective of this study was to determine if changes in PQC or ion transporter expression are correlated with survival of hypertonic environments by comparing the responses in two hypertonic environments. To date, published reports studied on salinity adaptation in euryhaline fish have focused mainly on ion-transporting mechanisms in gills [8]–[9], [38]–[39]. It was well established that the induction of branchial Na^+^/K^+^-ATPases (NKA), cystic fibrosis transmembrane conductance regulator (CFTR) and Na^+^/K^+^/Cl^–^ co-transporter (NKCC) in mitochondrion-rich (MR) cells are considerably important for hypertonic adaptation in various teleosts [8], [10], [14], [22]–[23], [25], [39]. When tilapia were transferred from FW to 20‰ SW, gill NKA activity elevated at 12 h post-transfer [16] while change in branchial CFTR localization was first observable at 24 h post-transfer (Fig. 2A-I). Since the antibody T4 has been demonstrated to recognize apical NCC and basolateral NKCC successfully in MR cells of tilapia [14], [24], a decrease in apical NCC was found within 24 h post-transfer (Fig. 2J-R). These data suggested that CFTR and NKCC mediated ion-secreting mechanism in tilapia gills would be fully activated later than 24 h after transfer to 20‰ SW. In addition, the density of type IV (i.e., ion-secreting type) MR cells in tilapia yolk-sac membrane significantly increased at 24 h after exposure to full-strength SW (32‰) [24]. These results imply that ion-transporting mechanisms are not the early osmotic stress response because increased abundances of ion and water transporter proteins are generally apparent after at least 12–18 h of exposure to hypertonic shock [15]. Although immunofluorescent staining is effective in examination of protein localization and is regularly used to investigate the working model of ionoregulation in fish gills [8]–[11], [13], we cannot ignore the detecting limitation of this approach and rule out more rapid activation of pre-existing transporter protein. Measurements of direct ion flux through fish gills, however, are technically demanding.

Diverse stresses can result in protein inactivation, unfolding or misfolding. In response to various adverse conditions, the mechanism of protein quality control (PQC) depends on molecular chaperones and proteases to maintain a proper balance between synthesis, maturation, repair and degradation of proteins and prevention of protein aggregation within cells [2], [4]–[5], [40]–[41]. Numerous studies have demonstrated the vital importance of PQC mechanisms in many organisms, including prokaryote, plants, free-living nematode, insect and mammals [2], [4]–[5], [28], [40]–[46]. Different responses induced by environmental stressors in aquatic organisms have been studied previously [6]–[7], [12], [15], [19]–[20], [47]. However, to our knowledge, no study addressed the pivotal role of PQC mechanism in aquatic organisms upon environmental stresses. It is thus significant to explore the PQC mechanism regulated by heat shock proteins (HSPs) and ubiquitination system in tilapia exposed to different levels of hypertonic shock. HSPs with large molecular weight (e.g., 70 and 90 kDa) may serve as biomarkers of non-specific stressors in a wide range of organisms because they are highly conserved among organisms of different taxa [48]. In this study, the expression levels of gill HSP70 and HSP90 were up-regulated as early as 3 h following the onset of 20‰ hypertonic stress (Fig. 3). The transcripts of tilapia gill HSP70 evidently increased at 2 h after exposure to 600 mOsm/kg hypertonicity [15]. These data indicate that molecular chaperones are induced during the first hour of hypertonic stress, presumably to refold and repair damaged proteins for counteracting harmful effects and performing an important cytoprotective function [1]–[2], [5], [49]–[50]. On the other hand, in eukaryotic cells, the damaged proteins are first marked by ubiquitin for degradation and digested into small peptides [4]. Therefore, ubiquitin-conjugated protein levels serve as a bioindicator of protein damage *in vivo* [19], [21], [46]. Western blot and dot-blot analyses were used to examine ubiquitin-conjugated protein levels in previous studies [19], [21], [46]. In order to evaluate the effect of hypertonicity on the response of ubiquitination system in tilapia gills, the levels of ubiquitin-conjugated proteins were determined by both approaches. Protein ubiquitination in gills increased at 24 h post-transfer in response to 20‰ hypertonicity (Fig. 4 and Fig. S1 in Supporting Information S1). In addition, hypertonic stress-induced ubiquitin-conjugated proteins were also found in *Caneorhabditis elegans* [46]. Collectively, these results suggest that changes in global protein ubiquitinylation are a fundamental feature of hypertonic stress in cells from diverse organisms [2], [4]. This study shows that tilapia survival of 20% SW is associated with activation of molecular chaperone and ubiquitination systems in gills. The activation of PQC mechanism prevents cell injury and accumulation of irreversible protein aggregation that occurs in response to diverse stresses [27]–[28], [46], [51]. This cytoprotective regulation is supported by the data presented in Fig. 7A, which showed that tilapia gills exhibited low levels of protein aggregation during exposure to 20‰ SW hypertonic challenge that did not significantly differ from control (0 h) values.

In contrast, the survival rate of tilapia dramatically dropped at 5 h after direct transfer from FW to 30‰ SW (Fig. 1). The PQC system was not activated under these conditions (Figs. 5, 6 and Fig. S2 in Supporting Information S1) and protein aggregation increased 2-fold at 3 and 4 h compared with the control group (0 h) (Fig. 7B). Failure to activate the PQC system could cause accumulation of aggregated proteins that interferes with normal cellular homeostasis and causes cell death [2]–[4], [41], [46], [52] and that is associated with many human diseases [43], [53]–[54]. Recently, similar biological events were reported in polar insect (*Belgica antarctica*) and *C. elegans* exposed to thermal and hypertonic shocks, respectively. Elevated protein aggregation levels were found when the organisms were cultured in high mortality conditions [28], [46]. Therefore, acutely elevated levels of protein aggregates in tilapia gills might result in cell death and further disruption of gill osmoregulatory function, eventually causing death. Moreover, the correlation between mass mortality and elevated levels of protein aggregation in gills were also found in medaka (*Oryzias latipes*) and sailfin molly (*Poecilia latipinna*), euryhaline teleosts with FW preference, which were transferred from FW to 30‰ hypertonicity (unpublished data).

Furthermore, the importance of gill NKA responses for salinity adaptation as well as determination of osmoregulatory status has received much attention in many euryhaline fish [10]–[11], [39], [55]. We hypothesize that damaged or denatured NKA could prevent activation of ion secretion. In order to assess if osmoregulatory function of tilapia was disrupted by 30‰ hypertonicity, detection of gill NKA abundance in aggregated protein fractions was analyzed by Western blot. The result showed a significant increase (approximately 2.8-fold) of gill NKA protein in aggregated protein fractions at 4 h post-transfer to 30‰ hypertonicity (Fig. 9). Because gill NKA was seriously damaged/denatured, osmoregulatory responses could not be triggered when tilapia were exposed to 30‰ SW. To our knowledge, this is the first time that damaged NKA has been measured in fish gills following a salinity challenge. Our findings provided a reasonable explanation for why tilapia plasma osmolality was higher than 450 mOsm/kg at 3 h after transfer to 30‰ SW [16] and subsequently died within 7 h of transfer (Fig. 1).

Many molecular adaptations of euryhaline fish to salinity changes have been determined [7], [10], [15], [39], [56]. The present study is the first to provide evidence for early activation of PQC mechanisms functioning to prevent hypertonicity-induced protein aggregation in aquatic animals. This response may be critical for preserving gill cell viability during the early stages of acclimation to a hypertonic environment so that ion transport function (e.g., elevation of NKA, NKCC and CFTR) can be activated during later stages. The vital significance of PQC in aquatic organism indicated the conservation of this mechanism across diverse taxa and broad evolutionary similarities in the stress response.

## Supporting Information

Supporting Information S1 **Figure S1, Dot-blot analysis of the levels of ubiquitin-conjugated proteins in gills of tilapia directly transferred from fresh water (FW) to 20‰ seawater (SW).** (A) Ubiquitin-conjugated protein levels were shown as relative values based on dot intensities. (B) Ponceau S total protein stain of blots was used as loading control. (C) The levels of ubiquitin-conjugated proteins increased significantly at 24 h post-transfer from FW to 20‰ SW. The asterisk indicated a significant difference (P<0.05) compared with the 0 h time-point using Dunnett’s test following a one-way ANOVA. Values are mean ± S.E.M (n = 5). **Figure S2, Dot-blot analysis of the levels of ubiquitin-conjugated protein in gills of tilapia directly transferred from fresh water (FW) to 30‰ seawater (SW).** (A) Ubiquitin-conjugated protein levels were shown as relative values based on dot intensities. (B) Ponceau S total protein stain of blots was used as loading control. (C) No significant difference was found in the levels of ubiquitin-conjugated proteins when tilapia were transferred from FW to 30‰ SW. Values are mean ± S.E.M (n = 5).
(DOC)
