# Supplementary material for: Early Response of Protein Quality Control in Gills Is Associated with Survival of Hypertonic Shock in Mozambique tilapia
Source: PLoS One. 2013 May 14;8(5):e63112. doi: 10.1371/journal.pone.0063112 (PMC3653892; doi:10.1371/journal.pone.0063112)
Supplement: Supporting Information S1 — Figure S1, Dot-blot analysis of the levels of ubiquitin-conjugated proteins in gills of tilapia directly transferred from fresh water (FW) to 20‰ seawater (SW). (A) Ubiquitin-conjugated protein levels were shown as relative values based on dot intensities. (B) Ponceau S total protein stain of blots was used as loading control. (C) The levels of ubiquitin-conjugated proteins increased significantly at 24 h post-transfer from FW to 20‰ SW. The asterisk indicated a significant difference (P<0.05) compared with the 0 h time-point using Dunnett’s test following a one-way ANOVA. Values are mean ± S.E.M (n = 5). Figure S2, Dot-blot analysis of the levels of ubiquitin-conjugated protein in gills of tilapia directly transferred from fresh water (FW) to 30‰ seawater (SW). (A) Ubiquitin-conjugated protein levels were shown as relative values based on dot intensities. (B) Ponceau S total protein stain of blots was used as loading control. (C) No significant difference was found in the levels of ubiquitin-conjugated proteins when tilapia were transferred from FW to 30‰ SW. Values are mean ± S.E.M (n = 5). (DOC) [file pone.0063112.s001.doc]

**Supporting information**

**Materials and Methods**

**Dot blot analysis**

Levels of ubiquitinated proteins in gills were measured using an immunochemical analysis modified from Todgham et al. [1]. Equal amounts of total protein (10 μg) from each sample were blotted onto pre-wetted nitrocellulose membrane (0.2 μm pore size) (Sartorius, Epsom, Surrey, UK) in triplicate by gravity filtration using a BioDot dot blotter (Bio-Rad, Hercules, CA, USA). Wells were washed twice with 200 μl of PBST and then heat-fixed at 65°C for 20 min. Then, the membrane was blocked in 5% (wt/vol) nonfat dried milk in PBST for 1.5 h. Following blocking, the membranes were washed three times in PBST (for 5 min each). The membranes were incubated at room temperature for 3 h with primary antibody (anti-ubiquitin antibody, Cell Signaling Technology) diluted in 1% BSA and 0.05% sodium azide in PBST, washed in PBST, and subsequently incubated at room temperature for 2 h with secondary antibody. The membranes were developed after incubation with the BCIP/NBT kit (Zymed). The developed membranes were photographed and imported as TIFF files. The immunoreactive signals were analyzed using a software package (MCID software). The results were converted to numerical values to compare the levels of ubiquitinated proteins of the immunoreactive signals.

**Figure legends**

**Figure S1. Dot-blot analysis of the levels of ubiquitin-conjugated proteins in gills of tilapia directly transferred from fresh water (FW) to 20‰ seawater (SW).** (A) Ubiquitin-conjugated protein levels were shown as relative values based on dot intensities. (B) Ponceau S total protein stain of blots was used as loading control. (C) The levels of ubiquitin-conjugated proteins increased significantly at 24 h post-transfer from FW to 20‰ SW. The asterisk indicated a significant difference (P<0.05) compared with the 0 h time-point using Dunnett’s test following a one-way ANOVA. Values are mean ± S.E.M (n = 5).

**Figure S2. Dot-blot analysis of the levels of ubiquitin-conjugated protein in gills**

**of tilapia directly transferred from fresh water (FW) to 30‰ seawater (SW).**

(A) Ubiquitin-conjugated protein levels were shown as relative values based on dot

intensities. (B) Ponceau S total protein stain of blots was used as loading control. (C)

No significant difference was found in the levels of ubiquitin-conjugated proteins

when tilapia were transferred from FW to 30‰ SW. Values are mean ± S.E.M (n = 5).

Figure S1


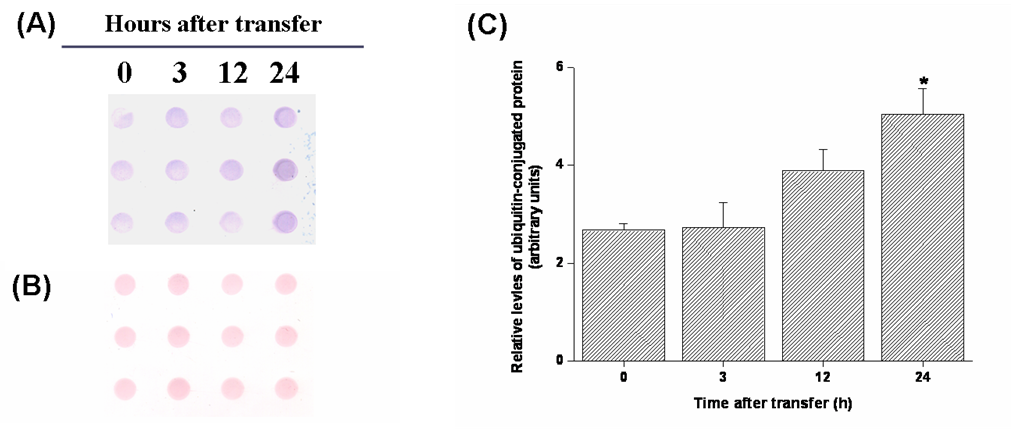


Figure S2


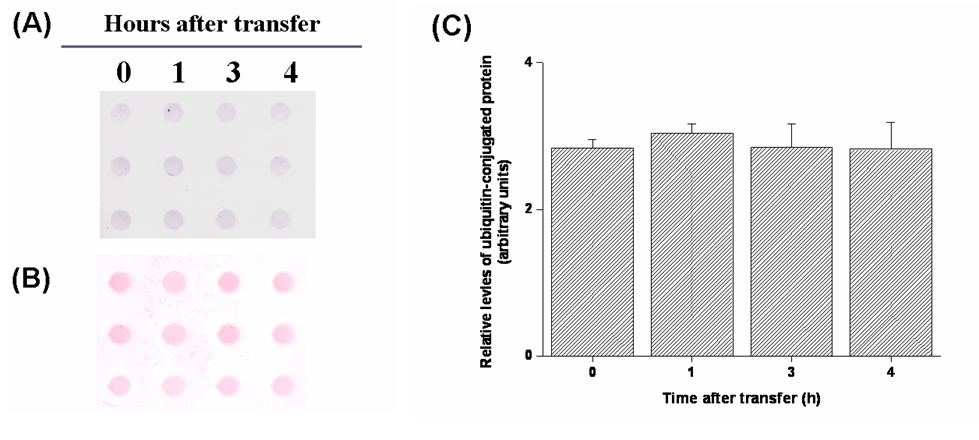


**Reference**1. Todgham AE, Hoaglund EA, Hofmann GE (2007) Is cold the new hot? Elevated
 ubiquitin-conjugated protein levels in tissues of Antarctic fish as evidence for
 cold-denaturation of proteins in vivo. J Comp Physiol B 177: 857-866.
